# Alternative Transcripts Diversify Genome Function for Phenome Relevance to Health and Diseases

**DOI:** 10.3390/genes14112051

**Published:** 2023-11-08

**Authors:** Shane A. Carrion, Jennifer J. Michal, Zhihua Jiang

**Affiliations:** Department of Animal Sciences and Center for Reproductive Biology, Washington State University, Pullman, WA 99164-7620, USA; shane.carrion@wsu.edu (S.A.C.); jennifer_michal@wsu.edu (J.J.M.)

**Keywords:** RNA variants, genome–phenome bridges, health and disease relevance, challenges and solutions

## Abstract

Manipulation using alternative exon splicing (AES), alternative transcription start (ATS), and alternative polyadenylation (APA) sites are key to transcript diversity underlying health and disease. All three are pervasive in organisms, present in at least 50% of human protein-coding genes. In fact, ATS and APA site use has the highest impact on protein identity, with their ability to alter which first and last exons are utilized as well as impacting stability and translation efficiency. These RNA variants have been shown to be highly specific, both in tissue type and stage, with demonstrated importance to cell proliferation, differentiation and the transition from fetal to adult cells. While alternative exon splicing has a limited effect on protein identity, its ubiquity highlights the importance of these minor alterations, which can alter other features such as localization. The three processes are also highly interwoven, with overlapping, complementary, and competing factors, RNA polymerase II and its CTD (C-terminal domain) chief among them. Their role in development means dysregulation leads to a wide variety of disorders and cancers, with some forms of disease disproportionately affected by specific mechanisms (AES, ATS, or APA). Challenges associated with the genome-wide profiling of RNA variants and their potential solutions are also discussed in this review.

## 1. Introduction

The number of genes in the human genome has been an open question in biology for decades. Historically, there were several significant waves of interest from the scientific communities, speculating answers to this question. The earliest recorded attempt can be attributed to James Spuhler, who in 1948 published an article titled “On the Number of Genes in Man”, where he proposed two estimates, 42 k genes, extrapolating from the chromosomal length of fruit fly genes, and 20–30 k, based on loci count derived from X-linked lethal mutations [1]. Vogel produced the next estimate in 1964 in “A Preliminary Estimate of the Number of Human Genes”. By assuming that the entire genome was protein-coding and genes were of comparable length (reasonable assumptions at the time), he used the molecular weight of hemoglobin to calculate the DNA weight of haploid chromosomes and divided it by his “standard” gene size, predicting an enormous 6.7 million genes, which he acknowledged seemed “disturbingly high”. He then posited that instead using the gene length of Dipteran giant chromosomes (~50 k nucleotides) would place the number of genes instead at 67 k, a number he was much more comfortable with [2].

The announcement of the Human Genome Project (HGP) appeared to re-galvanize interest in the subject, with a slate of papers on the subject being published in the 1990’s. The HGP was launched in 1990, with the goal of constructing the first full sequence of the human genome and identifying all protein-coding genes. The first prediction released by the Human Genome Project was 100,000 genes, driven by an assumption that that standard gene size was 30 kb, and was used as a baseline for many years afterwards [3,4,5]. The estimates that came after covered the gamut, from lows of 14,000 all the way up to 312,000, utilizing a variety of methods such as estimating from ESTs, chromosomes, and genome homologies [6,7,8,9]. Even as late as 2000, after the first rough draft of the genome assembly had been released, estimates varied significantly, from 26 k to 120 k, highlighting the difficulty of identifying protein-coding genes [10,11,12,13,14]. Though we now know the true number is likely close to 20 k, dependent on the stringency of filtering, it still fluctuates as our understanding evolves and more experimental evidence is found [15,16].

With that, the question became how our systems were able to develop and display such complexity with a protein-coding gene count (~20 k genes in 3000 Mb) a scant few hundred more genes than the simple nematode *C. elegans* (~19 k genes in 97 Mb) [7,17]. Human’s complex system requires a diversity of gene products to build and maintain the ~30 trillion cells through a rapid progression of tissue- and stage-specific proliferation, differentiation, and development [18]. Decades prior, the discovery of alternative splicing (AS), alternative transcription start (ATS) and alternative polyadenylation (APA) sites hinted at potential explanations. At the time of their discovery, however, their significance was not well understood. Most protein-coding genes possess a dominant isoform, the product most prevalent in cells and tissues across time points and development stages, but these mechanisms allowed the creation of alternative transcripts from these same genes [19].

For many years, AS was considered the dominant mechanism, contributing to transcriptome diversity and publications focused on the subject climbing 425% over 10 years, from 243 a year in 1990 when the HGP was announced to 1276 by the year 2000 with the completion of the first draft of the human genome assembly. Alternative splicing refers to a process where exons from the same gene are combined into different mRNA transcripts, allowing for multiple but related proteins with distinct structures and functions. Here we will refer to AS as alternative exon splicing (AES) to distinguish it from ATS and APA, which are also forms of alternative splicing. However, researchers have mounting evidence that AES is not the primary driver of transcriptome and proteome diversity, but our understanding of these processes (especially ATS and APA which have received relatively minimal attention comparatively) is still shallow [20,21].

Estimates for transcript variants have also ranged significantly over the years, from as low as ~46 k to over 300,000. Advancements in transcripts have led to more accurate gene databases (such as CHESS, Ensembl, Gencode, and RefSeq). Although the differences between them are shrinking, many discrepancies still exist [22,23,24,25,26]. The question we face now is how many transcripts have biological functions and how many produce a protein product? Here we review and summarize key findings about AES, ATS and APA site usages, exploring their prevalence, tissue use, motifs, and disease trends. We also discuss the challenges associated with RNA variant profiling, propose some solutions, and catalogue a significant number of experimentally verified isoforms and isoform functions from the available literature, comparing them to currently understood motifs. 

## 2. Alternative Exon Splicing (AES) Events: Features and Functions

### 2.1. AES—Increasingly Prevalent in Complex Organisms

Splicing (at the pre-mRNA stage, co- or post-transcriptionally) was discovered in an adenovirus experiment in 1977, which also discussed possible regulation of alternative splicing [27]. Alternative splicing was formally proposed as a theory by Walter Gilbert in 1978 [28]. The application of AES is influenced by the strength of the splicing signal, intronic/exonic enhancers or silencers, RNA binding proteins (RBPs), epigenetic modifications, genetic mutations and more. These factors can be fine-tuned by the organism to accommodate the development stage, differentiation, tissue/cell type, and other environmental elements [29,30]. AES sites are present in 95% of genes, with the average human gene producing three or more alternative transcripts, but is considered the least impactful in its contribution to RNA and protein diversity [20,21,30]. Despite this, its prevalence in higher eukaryotes has increased substantially from primitive eukaryotic organisms such as *C. elegans*, with an average of 6.4 exons per transcript compared to ~11 in humans. *C. elegans* also undergoes alterative exon splicing in only 25% of their protein-coding genes, indicating an evolutionary advantage to incorporating additional splicing elements [17]. 

There are currently five recognized forms of alternative splicing: exon skipping (aka cassette alternative), alternative 5′ and 3′ splice site within exons (where one side has a constitutive splice site and the other has 2+ alternative splice sites—meaning there are alternate regions that can be included or excluded), intron retention, and mutually exclusive alternative exons (two exons where one or the other, but not both, can be included) [31,32,33]. Exon skipping (~30%) and alternative 5′ or 3′ splice site within exons (~25%) account for most AES events in eukaryotes. Alterations to the mRNA may introduce premature stop codons (PTCs) resulting in truncated proteins, which frequently ends in decay of the RNA products through nonsense-mediated RNA decay (NMD) pathways. Multiple studies have shown the majority of alternatively spliced transcripts are either not expected to or do not produce a protein product or express it at such a minor level as to be undetectable using mass spectrometry [20,34]. These transcripts may be producing micropeptides (some new research suggests this may be the case), peptides transcribed from a short open reading frame (sORF) with a length of 100 or less amino acids (AA), or have regulatory functions as RNA [20,35,36]. Shorter isoforms are most often missing one or more exons (whole or partial), leading to potential domain loss, such as localization signals, regulatory domains, and binding sites [32]. 

### 2.2. AES—Dominantly Located in the Cytoplasm

Changes that lead to altered localization signals can affect the ability of an RNA or protein to be properly positioned, either by causing the transporter protein to be unable to dock or removing the localization signal entirely, as is the case in the isoform c-FLIP_S_. The *CFLAR* gene isoforms (called c-FLIP) are Death Effector domain containing proteins that are recruited to the DISC complex and regulate caspase-8 and 10 as well as DR5, playing a role in FAS-mediated apoptosis and necroptosis as well as T-cell proliferation. While the long form contains a catalytically inactive caspase-like domain that contains a nuclear localization signal, resulting in a large proportion of that isoform in the nucleus, the short form includes exon 7, which contains a stop codon. This truncated protein is missing the domain containing the localization signal and is restricted to the cytoplasm where it acts in an anti-apoptotic manner, as opposed to the long form that can be either pro- or anti-apoptotic in function [37,38]. 

*NUMA1* is another example of short isoforms localizing in the cytosol due to alternative exon splicing. The full (long) form of *NUMA1* is a large protein (~238 kDa) consisting of N and C terminal globular domains, with a long central coiled-coil domain, and acts as a structural hub in the nuclear matrix, interacting with microtubules and involved in the formation and positioning of mitotic spindles. The nuclear localization signal in its C-terminal region allows this isoform to perform its function. The short isoform, *NUMA1-s*, consists of only the N-terminal globular region of the long isoform, and though its function has been only marginally explored compared to the long form, it appears to have strong tumor-suppressing effects, inhibiting the proliferation of HeLa, heavily impeding the formation of cell colonies and suppressing the expression of *MYBL2*, a gene known for being overexpressed in the development of multiple cancer types [39,40].

Short isoforms also commonly have either an antagonist effect to the long isoform, as seen in prolactin receptors (*PRLRs*), or a complementary effect as displayed by the short form of *OPA1* [41]. *PRLRs* have short and long form isoforms, which can act as dominant negatives towards the other. This prevents excessive signaling of one form, with the short form operating different signaling pathways than the long form [42,43]. In the case of *OPA1*, which regulates mitochondrial stability and energetics, the long and short forms work together to balance function. The long forms are fusion-competent but poor at energetics whereas the short forms are competent at energetics and poor at fusion. The ratio of isoforms allows the fine-tuning of mitochondrial performance [44]. 

This is reinforced by the experimentally verified group of alternatively spliced isoforms we collected from the available literature. Out of the genes for which we cataloged splicing isoforms for, nearly all had verified localization of those isoforms, and the majority of those had verified “short” forms with shorter lengths than the canonical isoform. Of the ~75% genes whose isoforms had both verified localization and short forms, almost half of those short forms localized to the cytoplasm (*NUMA1-s*, *IGF-1ea*, *c-FLIPs/r*, and *CD33-s*). These isoforms showed high degrees of tissue specificity, concentrated primarily in brain and muscle tissues [Table S1].

### 2.3. AES—Commonly Expressed in Tissue-Specific Manner

AES is commonly tissue- and development-stage-specific, allowing myriad cell types to efficiently use their resources by fine-tuning expression. Tissue-specific AES events can make up as many as 65% of total splicing events, with the major transcript expressed varying in up to 60% of coding genes [45,46]. The prevalence of these events also differs by tissue, with splicing in nervous, muscle (particularly cardiac), testis and blood tissues comprising the majority, and these events often extending to the protein level [45,47,48,49]. Recent studies have shown the presence of microexons, exons comprising 1–9 AA, produced by splicing events in neuronal tissues involved in cell differentiation, synaptic function, and axon guidance. Found on surface-accessible domains, especially in charged regions, these microexons are located in close proximity to or overlapping protein domains, providing an additional level of regulation [50].

Besides being tissue-specific, AES events are often developmentally regulated. This makes them a key factor in highly region-specific cell differentiation and morphogenesis in multiple tissues such as embryonic neurons, spermatozoa, skeletal muscle myoblasts and stem/progenitor cells, among others. The precisely timed swapping of splicing regulators, and the dominantly expressed isoform, is integral for the transition from fetal tissue to adult tissue [51]. An example of this is the transition of dominant *PTBP1* expression to *PTBP2* expression during differentiation of progenitor cells into postmitotic neurons [47,48,52]. These changes can also differ between regions of the same tissues, as occurs with the gene *LIMK2*, a member of the LIM kinase (*LIMK*) family that regulates actin dynamics through cofilin phosphoregulation. *LIMK2* encodes two isoforms: *LIMK2a,* the primary isoform, which is expressed evenly through the brain, and *LIMK2b*, which is highly expressed in the thalamus and cerebellum [45,53,54].

### 2.4. AES—Highly Dysregulated in Neurological Diseases

While these mechanisms allow for great diversity in the transcriptome and proteome, their dysregulation can have serious health consequences. Mutations in ~50% of the known RNA-modifying enzymes have been linked to human disease [55]. Splicing defects can arise from mutations to splicing elements, which are present throughout the genes in large numbers and can result in the deletion or creation of splicing elements, or to the splicing machinery itself. They are highly associated with nearly every aspect of cancer development, developmental syndromes like Prader Willi, and degenerative diseases such as retinitis pigmentosa [56,57]. Alternative exon splicing-related diseases fall into two broad categories: mutations within the transcript itself and mutations within the splicing machinery or regulatory elements. Mutations anywhere within the ORF may lead to frameshifts that result in transcripts often consigned to NMD, while changes in the CDS (SNPs/INDELS) can also lead to changes in amino acid identity. Mutations in introns, the UTR (particularly those regions closest to the coding sequence) and exon/intron borders can alter splicing elements, potentially leading to deleterious transcripts/proteins [31,52,56,58]. Splicing errors in the 3′ UTR can affect the stability and translation efficiency of transcripts, creating imbalances that lead to disease [46,49,52,59].

As only ~10% of a gene is comprised of exon coding sequences and changes to coding sequences most often have inconsequential effects, it should come as no surprise that ~85–90% of disease-causing splicing errors occur outside of exon regions [20,60]. Errors in brain tissue-specific networks are responsible for several known neurological disorders such as autism spectrum disorder (ASD) [48,56]. Mutations in RBFOX proteins, which are local regulatory factors, subsequently cause the alternative splicing of *SHANK3*, *CACNA1C*, and *TSC2*, all of which are involved in ASD. In addition, the mis-splicing of microexons by Ser/Arg RBPs is known to be involved in ASDs. Splicing errors are also highly associated with various forms of cancer [57,60].

## 3. Alternative Transcription Start (ATS) Events: Features and Functions

### 3.1. ATS—Genomically Aligned by Sequence Structures, Clustering Patterns, and Promoter Motifs

As the name suggests, alternative transcription start events, first noted by Zitomer et al. in 1984, employ ATS sites and promoters in order to create transcripts with alternate first exons (AFE) or alter the length of the 5′ end [61,62]. As with AES, many factors contribute to transcription start site selection, including the presence or absence of motifs like TATA boxes, sequence structures, and internal ribosome entry sites (IRES), and a wide variety of transcription factors, including tissue and promoter exclusive factors [61,62,63]. The use of alternative transcription start sites can produce isoforms with different amino acid compositions, potentially altering function, or change the available regulatory regions of the 5′ UTR by adjusting its length. Genome-wide analyses have shown that 50% (though likely more, due to limitations of technology at the time of the studies) of human genes have at least two transcription start sites, with nearly five promoter peaks per gene on average [61,63]. 

Like AES, ATS events can be divided into several categories, based on the proximity of ATS sites, the levels of expression within a cluster of ATS sites, the location of the ATS site (3 or 5′) compared to the primary transcription start (PTS) site, and the proximity of the ATS sites to the PTS site. When compared by proximity, most ATS sites can be found in clusters within the core promoter region, with single or distal ATS sites the minority. These clusters are categorized by the level of transcription initiation relative to each ATS site. Clusters with more ubiquitous expression profiles across their start sites are broad, while clusters that show dominant expression from one start site are categorized as sharp or peaked. Peaked transcription start site (TSS) clusters show strong correlation with tissue-specific expression, while broad profiles show correlation with ubiquitous expression [63]. Initiations from ATS sites distant from the primary site tend to produce transcripts with AFE, while those in close proximity tend to produce transcripts with altered 5′ UTR lengths [62].

**Figure 1:**
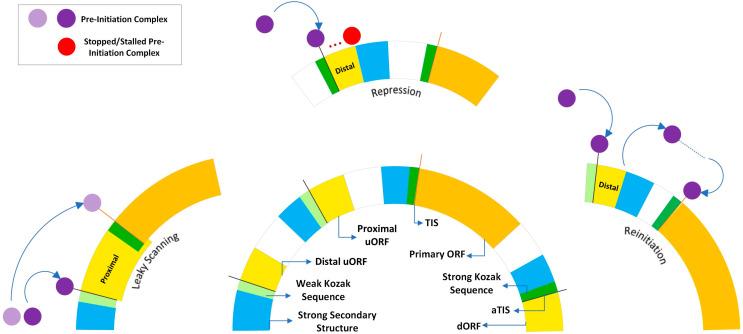
Three mechanisms impacted by ATS sites and ORFs. Leaky scanning can occur when translation begins at a start codon with a weak Kozak sequence, allowing a portion of the ribosomal subunits to skip over (leak) and initiate translation at an alternative start codon. Upstream ORFs and strong secondary structures from ATS sites/promoters can stop or stall the PIC complex, causing it to fall off and repressing gene expression downstream of it. Lastly, short uORFs separated from the main ORF can cause the ribosomal subunit to remain associated after termination and resume scanning, which is termed reinitiation. Abbreviations: ORF (open reading frame), dORF/uORF (downstream/upstream ORF), TIS (transcription initiation site), aTIS (alternative TIS).

ATS sites and promoters can also affect the translation of these transcripts, through strong secondary structures, IRES or the inclusion of additional upstream open reading frames (uORFs) through the extension of 5′ UTRs [64]. Translation can be initiated upstream or downstream of the primary ORF, which affects their translation level, Kozak sequence, start codon motifs and more. The Kozak sequence is highly important in start site selection, particularly the nucleotides at the −3 and +4 positions. While a start site with a strong Kozak sequence will primarily produce the dominant isoform, one with a weaker Kozak sequence will progressively utilize alternative start sites (also known as leaky scanning) [65,66,67,68]. Start sites that are upstream of the primary ORF and distal (not overlapping the primary ORF) are strongly correlated with short ORFs that can potentially encode small peptide products like micropeptides. This setup can also lead to the reinitiation of transcription further downstream, with the small ribosome 40 s subunit remaining associated with the mRNA after termination at the stop codon [65,68]. Both distal uORFs as well as proximal uORFs (which overlap with the primary ORF) instead often act as regulators of the primary translation initiation site (TIS), suppressing its activation, either partially or completely (Figure 1) [65,66,67]. 

Proximal ORFs often utilize non-canonical start codons, particularly CUG, with correspondingly weaker Kozak sequences, allowing for leaky scanning where some proportion of the ribosomal subunits fail to initialize at the start codon and continue scanning, allowing for multiple transcripts to be translated. Distal ORFs instead utilize more standard AUG codons, with strong Kozak sequences and secondary structures, in order to block or stall the transcription machinery before it can reach the primary TIS site [64,65,66]. Downstream ORFs also utilize stronger AUG codons and Kozak sequences in comparison to the primary TIS and are responsible for the N-terminal truncated proteins attributed to alternative start sites [66]. The most common ORFs are proximal to the primary TIS, acting in a repressive regulatory capacity [65]. 

### 3.2. ATS—Highly Involved in Altered N-Terminal Proteins, Localization, Stability, and Complementary Functions

ATS influences which uORFs are available in a transcript and as uORFs reside upstream of the canonical start site, most products will take the form of N-terminal extended transcripts or N-terminal truncated proteins, particularly for repressive uORF clusters. Extended N-terminal domains contribute to stability and translation efficiency without affecting the protein sequence [68]. This can be seen in the long isoform of *MAPKAPK2* (a gene whose primary isoform regulates the biosynthesis of pro-inflammatory cytokines), which uses a CUG start site in the 5′ UTR, displays markedly improved stability and is constitutively expressed [69]. 

ATS sites are also the primary means of changing isoform localization due to their manipulation of the N-terminal, the predominant location of localization signals. For example, in the gene *PTEN*, a well-known tumor suppressor with nuclear localization where *PTENα* and *PTENβ*, with extended N-terminal domains, localize in the mitochondria and the nucleolus, respectively. N-terminal truncated transcripts on the other hand can vary significantly, depending on the distance from the ATS sites to the canonical start site [70]. Close downstream start sites may yield products identical in function to the primary protein, while distant start sites produce proteins with different functions. 

An example of isoforms with divergent functions can be found in the isoforms of *ADK*, which acts as a sensor and regulator of the energy equilibrium in cell. The long form of *ADK* is prominent during early brain development, is nucleus localized, and is associated with the increased methylation of DNA and histones. The short form is prominent in the adult phase of brain development, particularly glial cells, and controls adenosine receptor activation [71]. 

Our collection of ATS site isoforms yielded a table of predominantly truncated isoforms. The localization of short and long isoforms differed in nearly every case (the few exceptions predominantly where the localization signal was not located on the N-terminal side, such as in *FRQ* in *Lachnellula* whose signal is in the C-terminal), though the location of the short isoforms varied. The majority of isoforms where specific tissues were noted were located predominantly in the brain, muscle, and heart tissues, with representation in liver, spleen, and pancreas. 

While no previous trend has been noted, in our dataset these isoforms demonstrated complementary instead of antagonistic functions, as in the genes *UL138*, *FRQ*, and *NR3C1*. For instance, both isoforms of viral UL*138* suppress immediate early (IE) gene transcription and generation of infectious CMV virions during latency. The long form is more effective at suppressing virion production during early stages, while the short form is more effective in later stages [72] [Table S2].

### 3.3. ATS—Frequently Tissue-Specific, Heavy Use of Intronic Enhancers 

Like AES, ATS sites show significant usage of tissue-dependent isoforms [20,63,64]. Transcription start sites show a degree of tissue preference in up to 80% of genes surveyed. Among protein-coding genes, 23% have two or more active promoters that contribute more than 10% of the gene’s expression. While most alterative promoters produce limited transcripts compared to the constitutively expressed primary isoform, there are a small percentage (~15%) where switches result in the alternative start site producing the dominant transcript [73]. The presence of CpG islands and absence of TATA boxes near and in promoter sequences are most associated with ubiquitous tissue expression and a tendency towards nuclear or mitochondrial proteins, whereas the opposite is found in tissue specific genes, with a tendency towards extracellular proteins [74]. 

Though our catalog of alternative start site isoforms shows a preference for the same tissues as alternative splicing, most of the literature discussing tissue specificity in ATS sites does not discuss specific tissue preferences, with the few exceptions pointing to higher cerebellar, muscle, heart, liver and testicular tissue use [20,75]. Intronic enhancers are common features in tissue-specific genes, and ~70% of enhancers in cardiac/muscle tissues mapped to the first intron [76]. Tissue-specific ATS sites are highly enriched in regulatory pathways of transcription and development, particularly along cell lines rather than cell types [64,77]. Distal upstream uORFs, upstream uORFs with AUG codons and optimal sequences, and secondary structures upstream of the canonical TIS all typically act as translational repressors, reducing the level of protein production. 

*PTPRJ*, which encodes a tumor-suppressing protein, utilizes alternate promoters with difficult-to-translate sequences, attenuating production [78]. This sort of regulation can change with external signaling, such as in ATF4, where the uORFs act in a repressive manner under normal conditions but become more permissive under stress conditions, allowing increased expression by the primary start site [35]. Several studies have noted increased activity of uORFs (particularly in response to eIF2α phosphorylation) during conditions of high stress that are highly conserved, indicating this is an evolutionary adaptation [68]. Alternative start sites also demonstrate temporal regulation, demonstrated by genes like *TEX101*, which produces a germ cell-specific protein involved in gonadal cells and is strongly involved in male fertility. While the first transcript is constitutively expressed in the gonads of both sexes, the second and third transcripts (which possess distinct 5′ terminal sequences) are expressed in males only after spermatogenesis [79]. More so than any of the other forms of regulation, uORFs have the capacity for the generation of regulatory micropeptides. This is demonstrated in *scl* in *Drosophila*, which encodes two micropeptides, each less than 30 AA, that regulate calcium transport impacting heart contraction [36].

### 3.4. ATS—Commonly Linked with Tumor-Specific Oncogenesis, Invasion, and Metastasis 

Errors in ATS sites and subsequent translation can lead to frameshifts or highly irregular amino acid conformations due to mutations. The silencing of ATS sites is also responsible for the expression of several diseases. While transcripts derived from ATS have had comparatively little research applied to their specific impact on the topic of disease association, studies show involvement in cancer phenotypes such as *CDC6* in breast cancer and *NRXN1* in neurological conditions [80,81]. Multiple types of associated diseases have been discovered in recent years that could be categorized as mutations within alternate ORFs, mutations that create new uORFs, aberrant promoter use, and change in imprinting status [67]. Oncogenesis and cancer progression are highly correlated with altered promoter use, shifting the transcript ratio to facilitate invasion, motility, metastasis, and more [82,83]. This deregulation of promoters is not only tissue-specific but tumor-specific, with different kidney tumors demonstrating different alternative promoter use [73]. The use of certain alternate promoters in *LEF1*, *TP73*, *NAT1* and other genes generate oncogenic transcripts. 

High levels of β-catenin/TCF complexes in colon cancer cells are capable of activating the promoter for the full-length transcript, setting up a positive feedback loop for WNT signaling, which is a hallmark of many colon cancers [83]. Mutations in the 5′ UTR can also result in translational errors. An example is the creation of a uORF in the human clotting factor 12 gene FXII, a coagulation protein. A single C to T SNP in the 5′ leader sequence results in the creation of a two-codon uORF, which also alters the strength of the Kozak sequence. While this change does not result in a change in mRNA levels it results in a marked decrease in protein expression, predisposing that individual to thrombosis [68]. Another example is a point mutation of G to T just upstream of the canonical start site in the gene *CDKN2A* (a strong tumor suppressor) creating a new AUG codon with a similar Kozak sequence, resulting in the primarily truncated gene product by effectively blocking translation of the canon AUG. The loss of this transcript results in increased motility, invasion, and metastasis in melanoma cells [61,82]. 

Finally, certain cancer lines can alter the imprinting status of genes, in particular genes with tumor-suppressor properties like *IGF2* and *PEG3*. In the case of *IGF2*, loss of imprinting allows the transcription of the normally silenced maternal allele, leading to the overexpression of IGF2 in some cancers such as bladder cancer [84]. Alternately, *PEG3* undergoes epigenetic silencing via hypermethylation of its promoters in many cervical and ovarian cancer lines, preventing transcription [85].

## 4. Alternative Polyadenylation (APA) Events: Features and Functions

### 4.1. APA—High Contribution to Transcript Diversity 

Alternative polyadenylation, discovered by multiple independent labs in 1980, has proven to be a major contributor to transcript diversity [86,87]. Polyadenylation site selection is a dynamic process, determined by predominantly cis elements, such as genetic motifs like the AAUAAA hexamer, the upstream UGUA, or downstream U/GU elements and their respective subunits APA site usage can be proximal or distal, with the former potentially changing the composition of the respective protein and distal usage conferring varying lengths of 3′ UTR [88,89]. APA usage is widespread, present in approximately 70% of 3′ UTRs in human genes, with ~50% of genes containing three or more polyadenylation sites [90,91]. This mechanism also appears to be highly conserved among eukaryotes, appearing in mammals, plants, and surprisingly even ~70% of yeast genes, which undergo nearly no alternative splicing, evidence of its evolutionary significance [92]. APA use is also prevalent in ncRNAs, with one genome-wide mouse study finding at least one significant APA isoform in ~79% of mRNA genes and 66% of lncRNA genes [93].

The existing literature has discussed different types of polyadenylation sites (PAS) utilizing a variety of nomenclature and schema, but here we categorize them broadly as tandem APA and upstream region (UR) APA. Tandem 3′ UTR APA occurs when both the proximal and distal APA sites reside within the 3′ UTR, changing the length of the 3′ UTR but leaving the gene product identical. UR-APA truncates the protein product to varying degrees and can be further classified as alternative last exon (ALE), intronic, or internal exons. ALE is the result of upstream splicing, resulting in a new terminal exon and PAS selection. Intronic APAs are utilized by bypassing or blocking of the 5′ splice site, causing an internal exon to extend into its adjacent intron. Finally, internal exon APAs are rare PASs that occur inside of an internal exon, producing a transcript with no stop codon and no 3′ UTRs [88,92]. The majority of APA PAS sites in multi-exon genes are tandem 3′ UTR, comprising approximately 67% of all PAS sites. Multi-UTR genes have markedly longer 3′ UTRs (nearly 4 × longer) than genes with single UTRs, with ubiquitously expressed genes exhibiting longer 3′ UTRs than tissue-specific genes, even longer than those expressed by neural tissues [94].

A few recent papers have suggested that the impact of APA on translation and stability are not as significant as previously thought; for example, finding APA site choice only influences ~10% of miRNA targeting [91,95,96]. Though these findings contrast with the previous literature, it should be noted that much of the regulation that occurs within 3′ UTRs is defined by regions (most notably AU-rich regions) that can be concentrated into adjacent motifs or spread throughout the entirety of the UTR, making identification difficult. On top of this, RBP binding sites can typically bind multiple different RBPs, denying easy regulatory identity, and even single RBPs have been shown to recruit different *trans*-factors (with a wide range of effects) when exposed to different stimuli [93,97]. Given confounding factors, including primary cell vs. cell line experimental setups, context-dependent results should be expected. 

**Figure 2:**
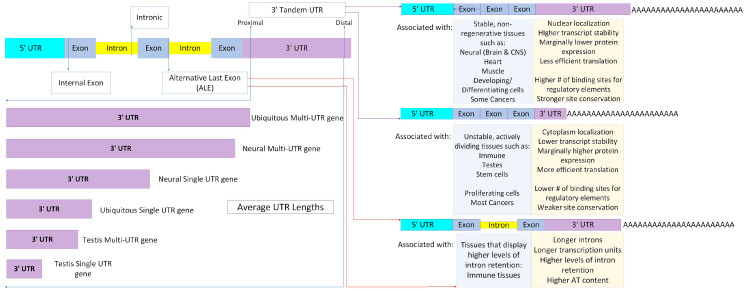
Different forms of polyadenylation, locations of typical polyadenylation signals (PASs) for each, and average lengths of untranslated regions (UTRs) based on tissue type and number of UTRs. The two most common forms, 3′ tandem UTR and alternative last exon (ALE), display common tissue associations and traits. The upper left shows the typical intragenic location of each form of alternative polyadenylation (APA) site. The lower left displays the average UTR length for single- and multi-UTR genes as well as ubiquitous versus tissue-specific genes. The right shows common associations for 3′ tandem UTR and ALE APA isoforms. Some ALE isoforms are the result of intronic APA site selection, as detailed on the right.

Collectively, UR-APA is significantly rarer than tandem, at up to 33% of total PASs, with ALE as the most frequent event, a coupling of alternative splicing and APA to produce an isoform with a different last exon and the use of an internal PAS [93]. As a result, ALE isoforms have different terminal coding sequences and 3′ UTRs. Intronic and internal exon APAs are the least common types of UR-APA, and have the highest probability of producing truncated proteins, with internal exon isoforms typically rapidly degraded by either the no-stop decay or nonsense-mediated decay pathways due to missing stop codons and/or UTR regions [88,91]. Evolutionarily, distal PASs are the most highly conserved, with strong consensus sequences and features while proximal PASs are poorly conserved between species, with weaker features [89,92]. PolyA signals have proven more similar in the same tissue across different species than for different tissues within that species [90]. Generally, longer isoforms tend to localize more to the nuclear fraction than the cytoplasmic one, with ~10% of all detected isoforms showing significant differences in nuclear/cytosolic abundance according to a recent study [89,91]. Overall, short isoforms are weakly correlated with higher protein production (without impacting mRNA expression), potentially due to their ability to more efficiently form polysomes. As in many cases with APA, however, it is highly context-dependent, with a small subset of short isoforms displaying marked increases in protein production of 40–100× (Figure 2) [98,99,100].

### 4.2. APA—Involved in Transcript Stability and Translation Efficiency

Tandem 3′ UTR modifications do not change the protein-coding sequence but still affect translation efficiency and localization through regulatory elements in their UTRs, including RBP binding sites, miRNA binding sites and scaffolding for RNA or protein transport [88,94,101]. As an example, *AAMDC-W*, an APA-derived isoform of AAMDC, is expressed at lower levels than both the L and S isoforms due to miRNA regulation [102]. Tandem 3′ UTR APA has also demonstrated the ability to regulate stability with 3′ UTR elements that contain destabilizing elements, such as miRNAs and RBPs that recruit decapping or deadenylating factors, and secondary structures that influence stability [88,94]. Isoforms of the gene *CALM1*, a calcium sensor and regulator, are an example of APA affecting stability. It expresses a short and long isoform, where the long form exhibits lower stability, with an expected half-life of ~50% of the short form [103].

Isoforms *RUNX1-A* and *CDC42(E6)* and *(E7)* are examples of ALE, having spliced alternate terminal exons and proximal PAS sites. *RUNX1A* is functionally antagonistic to its alternatively spliced isoforms, *RUNX1B/C*, balancing between differentiation and self-renewal in hemopoietic stem cells [104]. *CDC42*, a GTPase that regulates cell morphology and regulates multiple functions in the brain, has two isoforms called E6 and E7 [105]. The E6 isoform is both prenylated and palmitoylated—an indicator of strong membrane affinity. It is also brain tissue-specific and has mRNA localized to the soma, while the protein localizes to dendritic spines and plays a role in their formation. E7 is prenylated, giving it a hydrophobic c terminus, has mRNA localized to neurites and is expressed ubiquitously, while having a role in axonogenesis in neural tissues [76,77]. Several studies have suggested that non-coding transcripts generated by APA can act as scaffolding for the transport, production and regulation of other APA isoforms, particularly in the nuclear matrix [89,94]. The transmembrane *CD47*, which is associated with immune response, has a long and a short isoform. The long isoform contains a binding site for a complex of HUR-SET, which relocates it to the plasma membrane while the short isoform lacks these sequences and is localized to the ER [94]. 

Our collection of experimentally verified APA isoforms (where isoform tissue is noted) are all tissue-specific variants, with all but one expressed in brain tissues. The sole exception is *RUNX1A*, which is expressed in immature hematopoietic and progenitor cells. We found that in all but one case, short and long isoforms demonstrated different localization patterns, with long isoforms preferentially localizing to nuclei and to distal sites in neural tissues, whereas short isoforms preferentially localized to cytoplasm/cytoplasmic organelles and proximal sites in neural tissues [Table S3]. As with ATS sites, isoform function was overwhelmingly either complementary (as in *MCL1*) or, largely unique to APA, nearly identical but localized to a different tissue (as in *IMPA1*). The two isoforms of *MCL1*, *MCL1pa1* and *pa2*, show similar localization in the mitochondria, nucleus and cytoplasm, where they regulate apoptosis (anti- and pro-, respectively), mitochondria morphology, and cell proliferation but different translation efficiencies between the two isoforms keep the basal level of *MCL1* stable while allowing for quick adjustments based on cell requirements [106]. *IMPA1* on the other hand produces three isoforms, L/S/C, which localize to axons, but the L isoform enriches in distal axons where the S form enriches in proximal axons. In distal axons, a portion of the L isoform undergoes cleavage by an AGO2 complex to form the C isoform. All participate in the regulation of NGF-dependent pathways and are involved in the survival of neuron axons [107].

### 4.3. APA—Tissue-Specific Processes in Response to Proliferation and Differentiation 

Unsurprisingly, alternative polyadenylation has demonstrated significant tissue and temporal specificity in eukaryotes, with several clear motifs. There is clear evidence of tandem 3′ UTR and ALE regulation in neural, male and female reproductive, blood, muscle, stem cells and cancer tissues, despite the notable paucity of tissue-specific regulatory factors [91,92,108,109]. Tissue-specific regulation appears to rely on the prevalence/composition of core polyadenylation factors and competition with splicing factors (in the case of ALEs) in context-dependent fashion [91,93,109]. Globally, the enhanced use of proximal PAS is associated with proliferating cells where the use of distal PASs is linked to developing or differentiating cells [88]. This association extends to the respective tissues, with highly proliferative tissues, such as blood, showing overall preference for short isoforms, while more stable, non-regenerative tissues, such as heart tissue, show a preference for long isoforms [101]. 

The shorter, proximal PAS using form is also widely associated with cancers, though this has proven to be more a general association, with a selection of tissues and cancers (such as certain breast and thyroid) preferentially producing longer distal PAS-associated isoforms [88]. Thus, it may be more accurately stated that cancer cells display broad changes in PAS use, that can be proximal or distal dependent on tissue and cancer specificity. The best-known example of global APA regulation comes in neural tissues, where multiple studies have shown enriched translation of long 3′ isoforms, utilizing distal APA sites. In fact, neural tissue-specific genes demonstrate the longest 3′ UTRs out of all tissue-specific genes by a significant margin [94]. These isoforms show a preferred localization to dendrite and axon regions and, in several cases, demonstrate enzymatic cleavage of these long forms into shorter isoforms upon arrival. Shorter isoforms instead often localize to the soma but can be found elsewhere in the neuron, such as the axon, in response to stressors such as depolarization [107]. By contrast, hematopoietic cells (which have a high and constant rate of turnover) are known for their preference for shorter isoforms. Some of the oldest cells known to undergo APA are B cells, which produce an even ratio of the long and short heavy-chain isoforms in mature B cells, but shift progressively over to the short, secreted isoform in plasma cells as immunoglobulin secretion increases [88,100,110,111]. 

Initially, nearly all studies of polyadenylation focused on tandem 3′ UTR due to difficulties in identifying and isolating internal PAS usage, but in recent years several studies of ALE sites have emerged. Universally, these studies found ALE isoforms are regulated in a similar manner to tandem 3′ UTR isoforms in regard to PAS usage and the 3′ length in the tissues examined, with significant impacts on localization, and, in neural tissues, distal ALE isoforms preferentially residing in neurites [108,109,112]. They display longer introns, longer transcription units, and a higher AT content than other forms of APA, and tissues with higher ALE usage also demonstrate higher levels of intron retention, which suggests these factors play a part in their regulation. ALE isoforms are especially prominent in immune cells, and one study suggests that the prevalence of intron retention in blood cells provides the transcription complex the time to select ALE PAS sites, as happens in tandem 3′ UTR APA, and affects the selection of transcription start sites. An unexpected finding was that immune cells were enriched for intronic PAS in the 5′ end, producing transcripts that were less than 100 AA, indicating either non-coding or micropeptide transcripts [112]. Though the function of these transcripts is currently unknown, previous research suggests they may act as regulators themselves or act as scaffolding for the further regulation and production of APA as discussed prior [91,92]. This suggests similar findings might be made in other highly proliferative tissue types such as the gonads, particularly male gametes.

### 4.4. APA—Significantly Associated with Diseases in High Differentiation or Proliferation Profiles 

APA sites can affect amino acid composition by truncating mRNAs at various points, affect their localization, and even affect their stability and regulation. Mutations in APA sites are commonly linked to hematological diseases, certain cancers such as breast, brain, and colorectal cancers, immune syndromes like immune dysregulation, polyendocrinopathy, enteropathy, X-linked syndrome (IPEX), and neurological diseases [20,113,114]. Collectively, shifts in isoform production that result in the overabundance or minimization of an isoform can also result in disease states. We can categorize current disease causes as the loss or gain of individual APA sites and remodeling of APA site usage. Mutations as small as SNPs can introduce gain or loss of function in PAS, often the canonical PAS in the case of disease-causing mutations. Gain-of-function mutations, as seen in cases of systematic lupus erythematous (SLE), are the best known, with an A to G mutation in an alternate APA site leading to increased expression of a more stable form, resulting in increased levels in the cell. Individuals with this mutation appear to be susceptible to SLE [113,114]. 

Loss-of-function mutations are also prevalent, such as those that can occur in type 2 diabetes. A change in the C allele to a T allele in *TCF7L2* leads to the increased use of an intronic PAS that produces a truncated protein. This protein appears to repress TCF/LEF-dependent genes and is associated with increased risk for a particular form of diabetes [115]. The remodeling of APA site usage is commonly associated with cancer but is also the cause of several neurological and autoimmune disorders. Manipulation of APA regulators such as CSTF, CFI/CFII, and CPSF, pathways such as mTOR/Rho, and other factors such as PAP (poly(A) polymerase) are vital to the complex control of PAS site usage during normal development, and can contribute to the initiation or continuation of disease states when perturbed [89,91,92]. It has been proposed that short isoforms are favored in cancer due to the relative lack of RBP and miRNA binding sites, allowing them to escape regulation [91,92]. It is important to note, however, that different cancer lines can be highly variable, with different dependencies, sensitivities, and enriched pathways [88]. Thus, the effects of cancer on cell regulation have proven to be largely context-dependent, with several cancer lines preferentially expressing longer transcripts [91]. 

Studies of gastric cancer using the MKN28 cell line (regulated by the Rho GTPase pathway) have shown a switch in usage from the distal to the proximal PAS site, demonstrating strong transcriptional activity in the reporter gene, proving involvement in metastasis [88]. Conversely, studies in breast cancer using the MB231 cell line have shown many genes display preferential expression from distal APA sites, producing long isoforms [114]. Many of these are from genes associated with apoptosis and programmed cell death, indicating the use of the longer isoform may allow them to escape apoptosis of cancerous cells. Oculopharyngeal muscular dystrophy (OPMD) has been linked to a GCG expansion in the n-terminus of the *PABPN1* gene [88]. This mutated protein has been shown to sequester normal proteins in nuclear inclusions, instead of dispersing throughout the nucleoplasm like normal. This sequestering is common in neurological diseases, and this build-up of PABPN1 in nuclear inclusions has toxic effects [114].

## 5. AES, ATS and APA Events: Evidence of Cooperation and Antagonism

Though we have, to this point, discussed these three forms of modification as though they were largely independent, there is a significant amount of coordination, competition, and overlap between them. More than 80% of murine multi-transcript genes display interdependence between alternative splicing and the choice of transcription start site, with 37% of genes showing links between all types of features (TSS/Alternative exon, Alternative exon/APA, and TSS/APA) [116]. All three share regulatory factors, most significantly RNA polymerase II, particularly the C-terminal domain (CTD) of the largest subunit [32,117]. This CTD undergoes a number of modifications such as phosphorylation or methylation and can be further altered by cis/trans prolyl isomerases, which can alter its size and structure significantly and change which binding sites are presented and factor binding affinity [117]. As part of the complex, it recruits factors that help determine transcription start sites, splice sites, and polyadenylation sites used. 

U1 snRNP, one of the factors recruited by the RNA pol II CTD, impacts both splicing and polyadenylation. This protein suppresses premature 3′ end cleavage and polyadenylation, particularly in intronic cryptic PAS. Pushing the majority of PAS site usage to distal sites enriches primarily full-length transcripts in a process termed telescripting and lowers the proportion of transcripts being degraded. U1 snRNP also recognizes 5′ splice sites and base-pairing with the pre-mRNA, and is crucial for intron removal during splicing and alternative splicing [113]. Elongation or stalling of the pol II machinery has been associated with the selection of rarer AES sites (especially intronic), and the selection of proximal APA sites over stronger distal ones, while ATS-influenced translation reinitiation and leaky scanning rely on manipulating the PIC [59,65,115]. This can be caused by factors such as the secondary structure, chromatin state, histone modifications and a high AT or AU content, all of which can themselves interact with ATS, AES, and APA regulators [59,117]. Studies have shown that pausing of the PIC is more likely to occur at the uORFs of genes whose primary isoform is being repressed and near the proximal APA sites of highly expressed genes [115]. 

The extension of isoforms towards the 5′ or 3′ ends by ATS or APA provides more extensive regions for AES, particularly in the case of ATS. The selection of start sites impacts 5′ splicing patterns while promoter/enhancer activity can affect exon choice, including inducing exon skipping or the use of mutually exclusive exons. Coordination has been demonstrated between AES and APA in the cases of both tandem 3′ UTR and especially ALE, with the 3′ splice site and PAS communicating early in the transcription process, through PAS cleavage, the addition of the polyA tail, and concluding with splicing of the terminal exon [116,118]. 

An example of the coupled regulation of splicing and APA is represented in the gene *ACHE*. This gene, which terminates synaptic transmission, encodes multiple isoforms and is regulated by a combination of hnRNP H (another ribonucleoprotein) and CstF64. Here, hnRNP H causes distal PAS site use, inhibiting the selection of any proximal APA sites. Conversely, the inhibition of hnRNP H allows its antagonist CstF64 to bind intronic PAS sites, creating a truncated protein [110]. This short transcript can instead use the proximal 3′ APA site or retain intron 4. More generally, CstF64 acts in the same manner across a host of genes by binding intronic APA sites to activate their expression. CstF64 is, broadly, suppressed by U1 snRNPs, which, as previously discussed, are integral parts of the splicing machinery, recognizing 5′ splice sites [59]. 

The MBNL (Musclebind-like) family of proteins, regulators of RNA metabolism, is a known component of both the alternative splicing and alternative polyadenylation systems, particularly in muscle and neural tissues where it has been shown to bind splicing elements in nascent transcripts and 3′ UTR binding sites. Disruption of MBNL is highly associated with the altered localization and stability of transcripts and is a primary cause of the neuromuscular disease myotonic dystrophy (DM) [48,51,107,118]. The presence of co-transcriptional splicing has been proven experimentally, with spliced mRNA, spliceosome components, and splicing factors in the chromatin fractions (fragments created during the process of chromatin immunoprecipitation, more commonly known as ChIP) of actively transcribed genes before they are released into the nucleoplasm [119,120]. This was further confirmed in mammals, revealing that Ser/ARG-rich proteins (that bind the spliceosome), which would prevent hnRNPs (which transport pre-mRNA to degradation complexes) binding, were only effective when added before transcription. It was also shown that weakening of 3′ splice sites and inhibitory factors that bind intronic 3′ sites cause alternative splicing to occur post-transcriptionally instead of co-transcriptionally [119].

**Figure 3:**
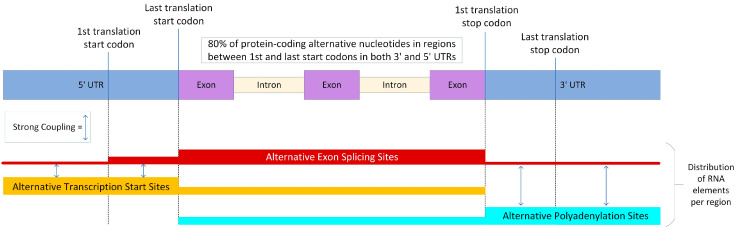
Distribution of post-transcriptional modification RNA elements. The layout of gene regions and distribution of the RNA elements within those regions for the three types of modification discussed here, alternative exon splicing, alternative transcription start sites, and alternative polyadenylation. It provides a visual representation of start rich and stop rich regions as the regions between the 1st and last start codon, and the 1st and last stop codon, respectively. It also denotes areas of coupling between these elements. Abbreviations: UTR (untranslated region).

The composition of these elements within the genes is an integral component of the complex interactions that allow for such a diverse transcriptome and proteome, with these elements falling into the 5′ UTR, coding sequence (CDS), and 3′ UTR areas. Of note is the region of the 5′ UTR between the first and last start codon, and the area between the first and last stop codon in the 3′ UTR, which for ease of use we will refer to as the start rich regions (STRR) and stop rich regions (SPRR). The elements of AS are most common in the CDS, STRR and 5′ UTR of genes, from highest to lowest. Their presence in the 3′ UTR and SPRR is low as these regions have minimal intron content. ATS elements are most common in the 5′ UTR and STRRs but still prevalent in CDS, while APA elements favor the 3′ UTR and SPRRs but are also prominent in the CDS (Figure 3) [21,58]. 

The intron content has proven to be a good indicator of polymorphic genes, which are more enriched for them than monomorphic genes. While they are especially prevalent in CDS, the STRR and SPRR also contain multiple introns, with the average being ~4 introns each. Correlations have been made between the number of introns and alternative nucleotide content, with more than 80% of protein-coding alternative nucleotides located in the STRR and SPRR in humans, and a similar 76% in the murine genome [21]. This placement shows the importance of these terminal extensions for transcript variance. Interestingly, tissue-dependent splicing is enriched amongst non-coding transcripts, and in non-coding exons generally. Combined with the weak expression of exons that display tissue-dependent splicing, only ~15% of this form of splicing is expected to involve primary transcripts, though this 15% could have significant impacts as they may proportionally produce altered proteins [20]. These data outline why AES is considered to have the least impact on transcriptome and proteome diversity, compared with ATS and APA. 

Broadly, highly expressed genes are intron-dense, and their distributions differ reliably, dependent on the functional area being examined (5′ UTR, CDS, etc.) [20,21,58]. N-terminal regions of proteins are enriched for intrinsically disordered regions (IDRs), which are segments with a higher proportion of charged amino acids and so lack a single unique 3D structure. This flexibility allows them to change conformation from an extended coil all the way to a collapsed globule (and any form in between) based on environmental contexts, allowing them to fulfill several purposes, like exposing and hiding motifs that mediate interactions with other proteins. Their adaptability makes them ideal regions for post-translational modification, facilitating substrate engagement and degradation, and for regulating binding partners by the adoption of different conformations. These IDRs, which are heavily involved in protein–protein interactions, are also enriched in alternative protein ends, suggesting more regulatory roles for proteins derived from alternative transcripts [121]. ATS and AES have shown strong coupling in 5′ UTR and STRRs, where AES occurs at a relatively high frequency but rarely without the co-occurrence of ATI. AES and APA also demonstrate strong coupling in the 3′ UTR but here they are not strongly correlated in SPRRs. Interestingly, the occurrence of AS in the coding sequence has shown a strong inverse relationship to the occurrence of AS or ATS in the 5′ UTR region [20]. On the other hand, AS in the coding sequence shows a positive relationship with APA events in the 3′ UTR and SPRRs. In fact, APA events occur in the 3′ UTR almost exclusively in the presence of AS in the CDS, further implicating the strong relationship between AS and APA [24,57,58,80].

## 6. Genome-Wide Profiling of RNA Variants: Challenges and Solutions

One challenge that is sometimes overlooked when considering the study of these mechanisms is the choice of appropriate tools. The massive influx of available RNA-seq data that accompanied next-generation sequencing methods and the increasing availability of long-read data from platforms like ONT and Pac-Bio have been accompanied by a proportionate increase in the release of detection and analysis tools designed to handle this data, many of which are tailored towards specific tasks. Hundreds of algorithms/potential pipelines exist for RNA-seq analysis, where the variability and quality of the data can range significantly. Each step of the process (trimming, alignment, counting, normalization, pseudoalignment (an alternative that combines alignment, counting and normalization into one step), and differential expression (DE)) has multiple tools available. Several benchmark studies have been conducted over the years, attempting to narrow down which tools produce the best combination of accuracy and precision. Though these studies could not find consensus, the general trend suggests that the selection of trimming and alignment algorithms were the least impactful, with counting and normalization being critical steps [122,123,124]. Despite this, the effects of normalization on the effectiveness of DE tools have been contested [125,126]. DE tools have shown high similarity in performance, with almost universally improved performance in precision, recall, and FDR as sample numbers increase [123,125,127]. One benchmark study found that exon-based methods (DEXSeq, edgeR, limma etc.) demonstrated higher precision and that exon- and event-based methods were generally low-FDR, high-precision and had moderate recall [128,129,130]. It was also found that the highest overlap of detected DE genes was among exon-based methods [131]. Several studies have suggested that DESeq2, limma and edgeR are overall reliable, non-biased, computationally light tools for differential expression [123,125,127,132]. 

As alternative splicing has long been considered integral to gene variation, it also possesses the most tools specific to the three mechanisms we have talked about in this paper. Unfortunately, benchmarking studies performed on these tools have suggested that the performance of individual tools is relatively unreliable, with high rates of false positives and a very low overlap of detected AES events between algorithms. Suggested means to combat this have included the use of more than one tool to increase the validity of the analysis or to utilize specific tools for the detection of particular types of events [133,134,135]. One study advised a combination of rMATS and Whippet, and provided a pipeline for users [135,136,137]. Recommendations for event-based tool selection varied from known annotated events (ASpli, Whippet, SGSeq) to intron retention (IRFinder) to de novo events (combination of tools like splAdder and Whippet/MAJIQ) [133,134,138,139,140,141,142]. Overall Whippet and rMATs are the tools most recommended for use in AS event detection, in combination with more specialized tools [133,134,135]. Tools specifically meant for AS detection/analysis utilizing scRNA-seq, like BRIE2, have not yet been benchmarked [143].

Tools for the detection and analysis of alternative transcription start sites and alternative polyadenylation sites are sparser than AES tools, though APA tools have been released with increasing frequency in recent years. For transcription start site detection and analysis CAGEfightR and TSRexploreR are known quantities, with SEASTAR, mountainClimber (RNA-seq data) and CamoTSS (scRNA-seq data) tailored towards alternative start site detection and usage rates [62,144,145,146,147]. We were unable to find any benchmarking studies for tools geared towards alternative transcription start sites. The development of APA detection and analysis tools has been prolific in the last decade, with algorithms covering the gamut from bulk RNA-seq to scRNA-seq, including machine learning and deep learning models. Many of these tools were developed for bulk RNA-seq, with the intention of leveraging the enormous stores of data in this format, but these tools consistently perform inferior to tools designed for 3′ seq or long-read (Pac Bio or ONT) data [148,149,150,151]. This is consistent with the difficulty and computational complexity of deriving APA sites and usage from data biased against 3′ ends. Benchmarking studies have found, like AES tools, that each algorithm returns highly individual APA results. These results have minimal overlap with each other, even amongst tools that utilize the same general method of detection (such as changes in read density as in TAPAS or APAtrap) and false positives are a common issue [148,149,151,152,153]. One study found that all RNA-seq input tools produced comparable numbers of APA sites, where sites found demonstrated the characteristics expected of polyA sites, while acknowledging the potentially high number of false positives [148]. A separate study promoted the use of ML and DL models for prediction from DNA, such as DeepPASTA, PASNet, or PASS [149,154,155,156]. The availability of annotations can play a significant role in the accuracy and precision of many of these tools, which often rely on databases for comparisons. All studies agreed that running any single tool was unlikely to perform for every task, with some suggesting a combination of tools, such as QAPA with a small number of Iso-Seq or 3′ Seq annotations to bridge the gap [148,149,151,157]. Other suggestions were picking the right tool for the job, such as TAPAS or DaPars2 for the de novo detection from RNA-seq data or APAtrap for plant data [149,151,158]. TAPAS, DaPars2, APAtrap, QAPA for bulk data and scAPAtrap for single-cell data were the most suggested tools in these studies [159]. An overall point of emphasis is that the use of any of these tools (AS or APA) requires independent validations of a random sampling of your findings [148,149,150,151]. Recommended tools can be found in Table 1. 

Many resources are integral to effective research, but few are as important as databases, particularly in motif or characteristic driven detection. As with tools, there are hundreds of databases, though many of them are no longer maintained or have been folded into more established databases. Several major databases contain variant transcript annotations, including Ensembl (ensembl.org, accessed on 1 November 2023), Gencode (gencodegenes.org, accessed on 1 November 2023), Genotype Tissue Expression portal (gtexportal.org, accessed on 1 November 2023) and NCBIs (https://www.ncbi.nlm.nih.gov/, accessed on 1 November 2023) Consensus Coding Sequence and RefSeq. Analysis of these database sequences forms the basis for many specialized databases, such as APAatlas, which was developed for the study of APA events in human tissues [165]. AES databases can be very specialized, from cataloging the effects of mutation on splice sites to clinical phenotypes to exon skipping [166,167,168]. ATS and APA databases are more generalized, documenting TSS and polyA sites, respectively, with some APA databases adding events and conservation, but the biggest variance is the species covered. A collection of some of these specialized databases can be found in Table 2. Many of the tools described above reference database annotations to locate events and sites, and accurate sequencing underpins all the discoveries in this paper, highlighting the importance of both improving current data and adding new sequenced genome data, samples, and tissues to expand our understanding and efficiency. 

A relatively recent category of tool–webtools sees sporadic entries. These are often aimed at enabling researchers with less bioinformatics or programming experience to take advantage of these advances. Though they lack the full flexibility of the modules they utilize, they offer ease of use with some flexible parameters, normally in a pipeline, leveraging multiple tools while mitigating the memory/cpu drain of running these same tools manually. These tools may also provide examples or previews of pipeline structures for a multitude of purposes. While webtools that are solely web-based only have limited functions, they provide a valuable resource suited for exploratory purposes and those with less programming experience. 

Finally, many methods have been developed to directly profile genome-wide expressed RNA variants, such as whole-transcriptome start and termini site-sequencing (WTSS-seq and WTTS-seq) methods [176,177]. These assays have advantages over the conventional RNA-seq. For example, WTSS-seq and WTTS-seq methods do not synthesize full-length cDNA, so there is no bias against long transcripts. The “5′ adapter—transcript target—3′ adapter” constructs are synthesized, avoiding the low-efficiency issues associated with ligation. There are no primer/template switches and/or changes in the protocol, which significantly minimizes amplification biases or detours. These assays involve just one run of PCR, thus minimizing the over-amplification of abundantly expressed transcripts and maximizing the transcriptome coverage. However, both WTSS-seq and WTTS-seq methods are tag-based approaches, so they cannot produce full-length transcripts. This should be easily overcome by using a long-read sequencing approach by linking the short tags to the full-length transcripts for functional characterization. 

## 7. Conclusions

Pre- and post- transcriptional modifications are extremely common and critical for transcriptome and proteome diversity. APA and ATS are responsible for most of this diversity, with AES’s contribution being widespread but minor. These phenomena utilize complex mechanisms and a variety of factors to alter the stability, location, and efficiency in a tissue- and stage-dependent manner. Some of these mechanisms and factors are unique to individual modifications but there is also overlap between them, notably the RNA polymerase II complex, where outcomes like proximal APA site selection benefit from pausing of the complex. These features can be antagonistic or cooperative, particularly between ATS and AES or APA and AES, influencing transcription start site and PAS selection. Collectively, dysregulation of these phenomena contributes to a large proportion of human disease, especially in neurological, blood, immune and muscle diseases, as well as various cancers. Given the integral role of these modifications in all levels of cell function, and their role in disease when dysregulated, it is important that we continue to study these phenomena. The study of these mechanisms requires careful consideration, the strengths of the tools selected need to match the aims of the research and often the use of more than one algorithm for detection and analysis is recommended. We have still only identified very limited numbers of their factors (ex: RBPs), their environmental context, or their networks (protein or otherwise). With improvements in sequencing techniques, identification methods, tissue profiling, and advancements in machine learning, we will be able to link these profiles to modifications, phenotypes, and clinical applications. 

## Figures and Tables

**Table 1:** Recommended method by mechanism. A list of tools recommended by benchmarked studies by purpose, name, programming language and article source [129,130,132,136,137,152,153,157,158,159,160,161,162,163,164].

Method Type	Method Name	Program Environment	Introductory Article
Recommended DE Tools	limma	Bioconductor R package	https://doi.org/10.1093/nar/gkv007, accessed on 1 November 2023
	edgeR	Bioconductor R package	https://doi.org/10.1093/bioinformatics/btp616, accessed on 1 November 2023
	DESeq2	Bioconductor R package	https://doi.org/10.1186/s13059-014-0550-8, accessed on 1 November 2023
Recommended AES Tools	rMATs	R package	https://rnaseq-mats.sourceforge.io/, accessed on 1 November 2023
	Whippet	Julia	https://github.com/timbitz/Whippet.jl, accessed on 1 November 2023
Recommended APA Tools RNA-seq	TAPAS	R package	https://doi.org/10.1093/bioinformatics/bty110, accessed on 1 November 2023
	DaPars2	Python	https://doi.org/10.1038/ncomms6274, accessed on 1 November 2023
	APAtrap	R package/PERL	https://doi.org/10.1093/bioinformatics/bty029, accessed on 1 November 2023
	QAPA	R package/Python	https://doi.org/10.1186/s13059-018-1414-4, accessed on 1 November 2023
Recommended APA Tools scRNA-seq	scAPA	R package	https://doi.org/10.1093/nar/gkz781, accessed on 1 November 2023
	scAPAtrap	R package	https://doi.org/10.1093/bib/bbaa273, accessed on 1 November 2023
Web Tools	eVITTA		https://doi.org/10.1093/nar/gkab366, accessed on 1 November 2023
	SpliceTools	PERL for download	https://doi.org/10.1093/nar/gkad111, accessed on 1 November 2023
	APAview	Jinja/Python	https://doi.org/10.3389/fgene.2022.928862, accessed on 1 November 2023
	Cas-Viewer		https://doi.org/10.1186/s12920-018-0348-8, accessed on 1 November 2023

**Table 2:** Databases by mechanism. A list of databases by primary mechanism, with database name, brief description, and url [166,167,168,169,170,171,172,173,174,175].

Database Type	Database Name	Description	Website
AES	MutSpliceDB	Effects of mutation on splicing	https://brb.nci.nih.gov/splicing/, accessed on 1 November 2023
	VastDB	Splicing in multiple species	https://vastdb.crg.eu, accessed on 1 November 2023
	HEXEvent	Human exon splicing	https://hexevent.mmg.uci.edu, accessed on 1 November 2023
	ExonSkipDB	Exon-skipping events	https://ccsm.uth.edu/ExonSkipDB/, accessed on 1 November 2023
	ClinVar	Variants with clinical phenotypes	https://www.ncbi.nlm.nih.gov/clinvar/, accessed on 1 November 2023
ATS	DBTSS	Human adult and embryonic tissues	https://dbtss.hgc.jp/, accessed on 1 November 2023
	refTSS	Human and mouse	https://reftss.riken.jp/, accessed on 1 November 2023
APA	PolyASite 2.0	Sites and usage in human, mouse and worm	https://www.polyasite.unibas.ch/, accessed on 1 November 2023
	PolyA DB3	Sites, cleavage, and conservation	https://exon.apps.wistar.org/PolyA_DB/, accessed on 1 November 2023
	scAPAdb	Sites and usage in multiple species, single-cell data	http://www.bmibig.cn/scAPAdb/, accessed on 1 November 2023

## Data Availability

All data generated or analyzed during this study are included in this published article and its Supplementary Materials (Tables S1–S3).

## Supplementary Materials

The following supporting information can be downloaded at: https://www.mdpi.com/article/10.3390/genes14112051/s1. Table S1: Alternate Exons Splicing Isoforms, Table S2: Alternative Start Site Isoforms, Table S3: Alternative Polyadenylation Isoforms. Refs [178,179,180,181,182,183,184,185,186,187,188,189,190,191,192,193,194,195,196,197,198,199,200,201,202,203,204,205,206,207,208,209,210,211,212,213,214,215,216,217,218,219,220,221,222,223,224,225,226,227,228,229,230,231,232,233,234,235,236,237,238] are cited in the Supplementary File.
