# Supplementary material for: Alternative Transcripts Diversify Genome Function for Phenome Relevance to Health and Diseases
Source: Genes (Basel). 2023 Nov 8;14(11):2051. doi: 10.3390/genes14112051 (PMC10671453; doi:10.3390/genes14112051)
Supplement: Supplementary file 1 [file genes-14-02051-s001.zip › genes-2687482 Supplemental update.pdf]

Table S1. Alternative Exon Splicing Isoforms. A collection of experimentally verified isoforms with isoform designation, relative length, localization, tissue studied, and function where found [37–40,42–44,48,54,102,104,178–185,187–201,203–213,222–223].

Gene	Isoform	Length	Localization	Tissue	Effect
AAMDC	-Q	Short	Not found	Not found	No detectable protein, possibly lncRNA
ATG4	A	Long	Not found	Ubiquitous	Contribute to residual priming activity, sufficient to enable lipidation of endogenous GABARAPL1 on autophagic structures. Processes LGG-1 100 fold more efficiently than ATG4B. Prefers GABARAP/GATE family to LC3
	B	Long	Not found	Ubiquitous	Required for priming of most LC3 subfamily isoforms (1500 fold more efficiently than any other isoform), efficient delipidation, main human isoform. Depletion shows severe but incomplete defect in processing/autophagy
	C	Short	Cytosol/Mitochondria (conditional)	Ubiquitous	Contribute to residual priming activity, sufficient to enable lipidation of endogenous GABARAPL1 on autophagic structures, less activity than ATG4A. Poor primers, efficient at delipidation.
	D	Short	Cytosol/Mitochondria (conditional)	Ubiquitous	Contribute to residual priming activity, sufficient to enable lipidation of endogenous GABARAPL1 on autophagic structures, less activity than ATG4A. Poor primers, efficient at delipidation. Affinity for damaged mitochondria, overexpression leads to apoptosis.
BDNF	Mature	Long	Dendrites and Soma	Brain	Linked to long term potentiation
	ProBDNF	Short	Soma	Brain	Determine neuronal fate, pathway of further development and differentiation
BRD4	-L	Long	Nucleus	Breast	Inhibits tumor progression, limits metastatic potential in certain tissues. Knockdown delays G1 cell cycle progression
	-S(a)	Short	Inner face of nuclear membrane	Breast	Promotes breast and ovarian cancer growth and metastasis, knockdown leads to S-phase arrest
CD33	-Long	Long	cell surface	Microglia	Associated with reduced phagocytosis in macrophages,

					causes susceptibility to Alzheimer's disease
	-Short	Short	cytoplasm	Microglia	Associated with reduced long form expression, increased phagocytosis, resistance to Alzheimer's disease
CDC42	(E6)	Short	Dendritic Spines	Brain	Prenylated and palmitoylated. Plays a role in dendritic spine formation, role in developing neural tube
	(E7)	Long	Axon compartment	Ubiquitous	Prenylated. Stimulates axon specification in dose dependent manner, role in developing neural tube
CEACAM	1-L	Long	cell-cell contact points	Liver/Colon	Associated with invasion, metastasis and recurrence in hepatocellular carcinoma, can act as tumor suppressor in other cancers. Promotes invasion and migration in colorectal cancer.
	1-S	Short	soluble, scattered over cell surface	Liver/Colon	Short form not associated with vulnerability
CFLAR	c-FLIP$_L$	Long	Nucelus(predominately)/cytoplasm/ ER/Mitochondria Associated Membranes	Ubiquitous	Can act in pro or anti-apoptotic manner. High levels correlate low expression of the short form, with resistance to fas-mediated apoptosis and necroptosis. Role in T cell proliferation.
	c-FLIP$_S$	Short	Cytoplasm	Not found	Short forms act in anti-apoptotic manner.
	c-FLIP$_R$	Short	Cytoplasm	Not found	Short forms act in anti-apoptotic manner. Titrates away and blocks CASP8
IGF-1	Eb	Long	Nuclear Fraction/ER	Muscle	Responsible for satellite cell activation and proliferation, both isoforms protect against age related muscle loss
	Ea	Short	Cytoplasm	Muscle	Sustains hypertrophy and regeneration in senescent skeletal muscle. Activates additional pathways such as AMPK, able to minimize oxidative damage. Promotes satellites cell differentiation and provides most of the mature IGF-1 for stimulating protein synthesis
NR3C1	hGRβ	Long	Nucleus/cytoplasm-translocates to nucleus	Ubiquitous	Natural dominant negative inhibitor of hGRα, can directly induce and repress a large number of genes not controlled by hGRα. Lower expression than alpha isoform except in epithelial and neutrophil cells.

	hGRα	Long	Nucleus/cytoplasm-translocates cytoplasm to nucleus	Ubiquitous	Bind glucocorticoids, recruits coregulators to exert transcriptional effects
NUMA1	-long	Long	Spindle/Nucleus	Ubiquitous	Mitotic spindle assembly/maintenance. Structural component of nuclear matrix.
	-short	Short	cytoplasm	Ubiquitous	Inhibit proliferation of HeLa cells, greatly restrain formation of cell colonies. Suppress expression of MYBL2. Greatly decreased expression of short form in gastric cancer tissues.
OPA1	-l	Long	Membrane anchored	Ubiquitous	Fusion competent for mitochondrial network morphology, not energetics competent, both types of isoform competent at maintaining cristae shape and junctions. Overexpression protects from muscular atrophy, ischemic damage and hepatocyte apoptosis.
	-s	Short	Mitochondrial intermembrane space	Ubiquitous	Efficient at preserving energetics (mitochondrial respiration and RCS organization), not fusion competent for mitochondrial network morphology, both types of isoform competent at maintaining cristae shape and junctions
PKM	1	N/A	cytoplasm/nucleus	Brain/Muscle/Heart-somatic tissues	Used in TCA cycle, glucose metabolism, possible implication of tumor involvement in certain cancers
	2	N/A	cytoplasm/nucleus	Embryonic, proliferating, and cancer cells	Glucose metabolism, more favorable to aerobic glycolysis. Promotes Warburg effect
PRLR	-Long	Long	Not found	Ovary/Uterus/Breast, most other tissues lesser	Can act as dominant negative to the other isoform and prevent excessive signaling of one isoform. Requires both isoforms for corpus luteum survival.
	-Shorter	Short	Not found	Kidney/placenta/pancreas/liver	Can act as dominant negative to the other isoform and prevent excessive signaling of one isoform. Requires both isoforms for corpus luteum survival. Operates different signaling pathways than the long isoform in PDAC cells, acts a tumor suppressor.
RUNX1	A	N/A	Not found	Immature hematopoietic stem and progenitor cells	Overexpression expands functional HSCs in vitro and in vivo, retards hematopoietic differentiation, and enhances engraftment of murine bone marrows cell after transplant.

					Overexpression associated with leukemias
	B/C	N/A	Not found	Not found	Promote differentiation and abrogate engraftment of transplanted murine bone marrow cells
STIM1	L	Long	Not found	Muscle	Allows immediate activation of SOCE, required to trigger repetitive cytosolic Ca2+ releases. Can permanently cluster.
	S	Short	ER (predominately)/Plasma membrane	Ubiquitous	ER Ca2+ sensor controlling SOCE and CRAC channels in non-excitable cells.
	A	Short	ER	Astrocytes, heart, kidney, testes	Dominant negative regulator of SOCE and I_{crac}, facilitating sequence-specific fast calcium dependent inactivation and destabilizing gating of Orai channels
	B	Unclear	Presynaptic ER	CNS/Cerebellum	Increases vesicle release at high demand, alters slow Ca2+ dependent inactivation of I_{crac}
FYN	B	N/A	Not found	CNS	Less efficient phosphorylation of Sam68 than FYNT-change of SH2 inase linker onto both SH3 and kinase domains. Decoupled from apoptosis regulation in epithelial cells
	T	N/A	Not found	Hematopoietic	Specific regulation of Sam68 dependent BCL-X alternative splicing and cell apoptosis
FAK	+6,7	N/A	cytoplasm (cytoskeleton)	CNS (Neuron/glia)	Enhanced autophosphorylation, strong intramolecular phosphorylation
	+3	N/A	Not found	Not found	Same low autophosphorylation rate as primary isoform, undergoes intermolecular phosphorylation in intact cells
	+6,7,28	N/A	Not found	CNS	Enhanced autophosphorylation, strong intramolecular phosphorylation
QKI	-5	N/A	Nucleus	Not found	Inhibits cell proliferation and migration (regulates splicing of NUMB exon 12, competes with SF1 at branchpoint)(ADD3 exon 14, binds 3' region of intron 13). Inhibits miRNA transcription and transportation. Highest during embryogenesis and declines with age.
	-6	N/A	Cytoplasm/Nucleus (Shuttle)	Not found	Inhibits miRNA transcription and transportation. Expression during late embryogenesis and peak

					coincides with myelination in mouse brain. Positive regulators of oligodendrocyte and Schwann cell differentiation, myelin production.
	-7	N/A	Cytoplasm	Not found	Expression during late embryogenesis and peak coincides with myelination in mouse brain. Positive regulators of oligodendrocyte and Schwann cell differentiation, myelin production.
NTRK2	A (trkB-fl)	Long	Neurons	Brain	Transmembrane protein, BDNF binding signals PI-3K and MAP kinases. Promotes neurogenesis
NTRK3	β (trkB-t)	Short	Astrocytes	Brain	Transmembrane protein, highly expressed during neurogenesis to gliogenesis transition, directs differentiation into glial cells. Can antagonize effects of BDNF on neuronal survival, modulate neurite outgrowth, and activate calcium signaling pathways. Highly expressed in gliomas
ROBO	1	N/A	Not found	Embryonic tissues, brain/eye/kidney in adult	Transmembrane protein, interacts with extracellular SLIT. Higher expression in neurons, expressed by most pioneer axons. Associated with spatial regulation in nervous system.
	DUTT1	N/A	Not found	Ubiquitous	More highly expressed in development. Transmembrane protein, interacts with extracellular SLIT. Main form expressed in spinal cord commissural neurons. Associated with lung development and tumorigenesis
ROBO3	A	N/A	Not found	Several, high in Brain	Incapable of binding Slit, regulated through PI3-kinase. Might have significant role in the brain. Expressed in commissural neurons, suppresses Slit-mediated repulsion
	B	N/A	Not found	Not found	Binds the ROBO ligand Slit, regulated by serin/threonine protein kinase. Upregulated in RASF and melanoma.
LIMK2	A	Long	Cytoplasm/Nucleus (Shuttle)	Thymus, spleen, liver, intestine (preferred)	Modulated G2/M arrest. Role in microtubule organization. Phosphorylate cofilin, including leading to remodeling actin cytoskeleton during neuronal differentiation.
	B	Short	Cytoplasm predominantly	Brain, lung, heart, kidney, testis	Upregulated by P53, modulated G2/M arrest. Role in microtubule

					organization. Phosphorylate cofilin, including leading to remodeling actin cytoskeleton during neuronal differentiation.
AGAP2	PIKE-L	Long	Cytoplasm/Nucleus	Brain (absent cerebellum)	Forms complex with mGluR I-Homer to activate PI 3-kinase activity and prevent neuronal apoptosis
	PIKE-A	Short	Cytoplasm/Nucleus	Ubiquitous	Regulates Akt/PKB activity, associated with cancer cell migration
	PIKE-S	Short	Nucleus	Brain	Acts as a nuclear GTPase, activates PI 3-kinase
RBFOX1	Δ19	Short	Nucleus	Not found	Regulation of splicing networks
	-L	Long	Cytoplasm	Not found	3' UTR binding and competition with other factors (like miRNAs) to stabilize synaptic and autism related genes

Table S2. Alternative Start Site Isoforms. A collection of experimentally verified isoforms with isoform designation, relative length, localization information, tissue studied, and function where found [69–72,80–81,104,186,189–192,207–209,212–229].

Gene	Isoform	Length	Localization	Tissue	Effect
AGAP2	PIKE-L	Long	Cytoplasm/Nucleus	Brain (absent cerebellum)	Forms complex with mGluR I-Homer to activate PI 3-kinase activity and prevent neuronal apoptosis
	PIKE-A	Short	Cytoplasm/Nucleus	Ubiquitous	Regulates Akt/PKB activity, associated with cancer cell migration
	PIKE-S	Short	Nucleus	Brain	Acts as a nuclear GTPase, activates PI 3-kinase
ROBO	ROBO1	N/A	Not found	Embryonic tissues, brain/eye/kidney in adult	Transmembrane protein, interacts with extracellular SLIT. Higher expression in neurons, expressed by most pioneer axons. Associated with spatial regulation in nervous system.
	DUTT1	N/A	Not found	Ubiquitous	More highly expressed in development. Transmembrane protein, interacts with extracellular SLIT. Main form expressed in spinal cord commissural neurons. Associated with lung development and tumorigenesis
ROBO3	ROBO3A	N/A	Not found	Several, high in Brain	Incapable of binding Slit, regulated through PI3-kinase. Might have significant role in the brain.

					Expressed in commissural neurons, suppresses Slit-mediated repulsion
	ROBO 3B	N/A	Not found	Not found	Binds the ROBO ligand Slit, regulated by serin/threonine protein kinase. Upregulated in RASF and melanoma.
ADK	ADK-L	Long	Nucleus	Heart/Spleen/Muscle	Prominent during development in brain. DNA and histone methylation. Bidirectional promoter
	ADK-S	Short	Cytoplasm	Brain	Prominent in adult phase of brain development. Astrocyte adenosine flow (adenosine receptor activation).
CASP-2	L	Long	Cytoplasm (predominately)	Ubiquitous	Much stronger promoter to control ratio, overexpression induces programmed cell death, decreased expression delays apoptosis in response to various stimuli
	S	Short	Nucleus (predominately)	Heart/Brain/Skeletal Muscle (contested)	Overexpression could suppress mammalian cell death, inhibits apoptotic blebbing and body formation. May bind and inhibit CASP-2L.
CDC6	-L	Long	Not found	Breast	Proto-oncogenic, contributes through its role in DNA replication, initiation and repressive effect on INK4/ARF locus.
	-S	Short	Not found	Breast	Upregulation correlated with increased protein levels and BrdU incorporation. Higher translation, possible increased S phase entry.
MYC	1	Long	Not found	Ubiquitous	Overexpression inhibits growth of COS cells. Preferred as cells approach high density growth arrest. Efficient apoptosis control
	2	Long	Not found	Ubiquitous	Predominant in growing cells. Competent in inducing cell growth, attenuated for apoptosis
	S	Short	Not found	Ubiquitous	Rescues viability and normal proliferation can't induce apoptosis.
FRQ	L	Long	Nucleus/cytosol	Not found	Ratio of both fine tune period length in response to ambient temperatures, overall levels crucial for amplitude of circadian oscillations, this isoform supports a shorter rhythm.
	-S	Short	Nucleus/cytosol	Not found	Ratio of both fine tune period length in response to ambient temperatures, overall levels crucial for amplitude of circadian

					oscillations, this isoform supports a longer rhythm. Splicing dependent on light/temperature
IGF-1	Ea	Short	Cytoplasm	Muscle	Sustains hypertrophy and regeneration in senescent skeletal muscle. Activates additional pathways such as AMPK, able to minimize oxidative damage. Promotes satellites cell differentiation and provides most of the mature IGF-1 for stimulating protein synthesis
	Eb	Long	Nuclear Fraction/ER	Muscle	Responsible for satellite cell activation and proliferation, both isoforms protect against age related muscle loss
MAVS	FL MAVS	Long	Mitochondria/MAM/Peroxisomes	Ubiquitous	Regulates production of type I IFN, STAT1
	miniMAVS	Short	Mitochondria	Ubiquitous	Suppresses production of type I IFN, STAT1
MAPKAPK2	Long	Long	Nucleus/cytosol shuttling	Not found	Identical function (so far) to canon isoform. Role in inflammation, stress signaling, and acts as a checkpoint kinase in cancer. Appears to have different substrate specificity.
	Short	Short	Nucleus/cytosol shuttling	Not found	Induce immediate early gene early growth response 1 (EGR1), cFos, and TTP. Involved in cell migration. Associated with migration associated and transcription regulation proteins.
NR3C1	hGRα-A	Long	Cytoplasm/Nucleus	Ubiquitous	High affinity to glucocorticoids, interaction with GREs following ligand activation. Regulate both common and unique sets of genes. Predominant isoform with B variant
	hGRα-B	Short	Cytoplasm/Nucleus	Liver/Thymus	1.5 times more effective in transactivation from a single GRE driven reporter gene than the alpha variant, transrepression remains the same. Predominant isoform with A variant
	hGRα-C1,C2, C3	Short	Not found	Pancreas/Colon	Enhanced activity in induction of proapoptotic genes, more efficient than D isoforms at recruiting coactivators. Most sensitive variant to cell killing effects of glucocorticoids.

	hGRα-D1,D2,D3	Short	Nucleus	Spleen/Lungs	Increased expression in schizophrenia patients, low activity in induction of proapoptotic genes. Exhibit constitutive binding to certain GRE containing promoters. Most resistant variant to cell killing effects of glucocorticoids.
NRXN1	-α	Long	Not found	Brain	Development of GABAergic synapses. Elevated expression in bipolar/schizophrenia.
	-β	Short	Not found	Brain	Selective binding of glutamatergic postsynaptic partner (Neuroligin-1, LRRTM1). Elevated expression schizophrenia.
P53	Δ40	Short	Not found	Not found	Balances tissue regeneration and tumor suppression while fine tuning p53 activity. Possible role in melanoma development.
	Δ133	Short	Nucleus (predominately)	Not detected in prostate, uterus, skeletal muscle, and breast	Inhibits p53 transcriptional activity, inhibits replicative senescence and promotes cellular proliferation of fibroblasts. Prevents p53 mediated apoptosis. Highly prone to aggregate.
	Δ160	Short	Not found	Not found	Possible role in mutant p53 gain of function. Apoptosis inhibiting.
PTBP3	FL	Long	Nucleus	Not found	Only 10% of transcripts, regulation of FAS splicing
	Δ2 AUG4	Short	Cytoplasm/Nucleus	Not found	Most abundant, not NMD sensitive, regulation of FAS splicing
	Δ4 AUG11	Short	Cytoplasm/Nucleus	Not found	Not NMD sensitive, less efficient due to poor localization, regulation of FAS splicing, RNA binding not impaired despite truncation of RRM
PTEN	α	Long	Mitochondria	Ubiquitous	Functions in mitochondrial metabolism
	β	Long	Nucleolus	Ubiquitous	Negatively regulates rDNA transcription and cellular proliferation
RUNX1	b	Long	Nucleus	Myeloid cells	More active at upregulating GM-CSF and Ialpha1 promoters.
	bΔN24	Short	Nucleus	Myeloid cells	Protein more stable, retains ability to promote hematopoiesis. Similar activity to inhibit growth of CB cells. Lower transcriptional activity
	B/C	Short	Not found	Not found	Promote differentiation and abrogate engraftment of transplanted murine bone marrow cells
TDP2	-L	Long	Mitochondria-nucleus/cytoplasm	Not found	Protect against DNA damage caused by TOP2 poison, mtDox,

					lack of leads to reduced mitochondria transcription
	-S	Short	Mitochondria-cytoplasm	Not found	Protect against DNA damage caused by TOP2 poison, mtDox, lack of leads to reduced mitochondria transcription
UL138	pUL138-L	Long	Golgi/cytoplasm	Not found	Suppress major immediate early (IE) gene transcription and generation of infectious virions in cells with HCMV. More potent at restricting virion production at early stages.
	pUL138-S	Short	Golgi/cytoplasm	Not found	Suppress major immediate early (IE) gene transcription and generation of infectious virions in cells with HCMV. More potent at restricting virion production at later stages.

Table S3. Alternative Polyadenylation Isoforms. A collection of experimentally verified isoforms with isoform designation, relative length, localization, tissue studied, and function where found [94–102–104,106–107,179–180,185,202,230–238].

Gene	Form	Isoform	Localization	Tissue	Effect
CALM1	CALM1-L	Long	Primary hippocampal processes	Neural tissues	Absence exhibits disorganized DRG migration in embryos and reduced experience induced neuronal activation in adult hippocampus. Role in central and peripheral nervous system. Less stable than short isoform.
	CALM1-S	Short	Primary hippocampal processes/DRG axons	Ubiquitous	
MCL1	pa1	Short	Mitochondria, nucleus/cytoplasm		Depletion causes severe defects in mitochondria morphology, increases apoptosis, and impacts cell proliferation. Increased expression on T cell activation, correlating with increased MCL1 protein levels. Responsible for anti-apoptotic function.
	pa2	Long	Mitochondria, nucleus/cytoplasm		Depletion causes severe defects in mitochondria morphology, increases apoptosis, and impacts cell proliferation. More expressed isoform but at lower efficiency (repressed by miR17). Pro-apoptotic. May provide basal level of MCL1.

RUNX1	A	Short	Not found	Immature hematopoietic stem and progenitor cells	Overexpression expands functional HSCs in vitro and in vivo, retards hematopoietic differentiation, and enhances engraftment of murine bone marrows cell after transplant. Overexpression associated with leukemias
AAMDC	-S	Short	Not found	Not found	Increased expression level and transcription efficiency. Overexpression enhanced lipid accumulation and expression of adipogenic marker genes in preadipocytes
	-W	Long	Not found	Not found	Increased expression level and transcription efficiency at a lower level compared to the short isoform due to miRNA regulation. Overexpression enhanced lipid accumulation and expression of adipogenic marker genes in preadipocytes at a lower level than the short isoform.
CDC42	E6	N/A	Dendritic Spines	Brain	Prenylated and palmitoylated. Plays a role in dendritic spine formation, role in developing neural tube
	E7	N/A	Axon compartment	All	Prenylated. Stimulates axon specification in dose dependent manner, role in developing neural tube
IRF5	V1	Short	Not found	Not found	Constitutively active, further stimulated in virus infected cells
EEF1A	1	N/A	Dendrite tips/spines	Ubiquitous	Endogenous were upregulated by type I lfns, V3 promoter responds to Ifn. Overall higher expression than A2 variant in dendrites, might coordinate remodeling of neuronal cytoskeleton. Higher affinity for GTP and eEF1B.
	2	N/A	Soma	Brain, heart, skeletal muscle	When upregulated, takes over protein synthesis in specific tissues for EEF1A1. Stimulate phospholipid signaling and activates AKT-dependent cell migration and actin remodeling. Favors oncogenesis. Expression increases over neurodevelopment. Capacity for co-translational targeting of nascent peptides for degradation under stress conditions. Balances actin bundling and local translation elongating in dendritic spines.

DNMT3A	1	Long	Embryonic stem cells, bivalent CpG island shores.		Responsible for maintaining methylcytosine turnover at bivalent CpG islands. Preferential localization to sites enriched for H3K27me3. De novo methylation.
	2	Short	Euchromatin	Not found	Localization to actively transcribed genes enhanced by H3K36me3. Mainly expressed in early development.
MEMO1	-Long	Long	Not found	Cerebellum	Becomes primary form during differentiation, long UTR acts as dock for miR-124, downregulating expression during granule cell differentiation
	-Short	Short	Not found	Cerebellum	Granule cell precursors only express this short form.
DSCAM1	-L	Long	Not found	Brain (Neurons)	Elav promotes this isoform. Knockdown results in severely compromised locomotion and adult lethality. Severely impaired mushroom bud bifurcatin and suppressed axon outgrowth of small ventral lateral neurons. Preferentially excludes exon 19 (regulated by Elav). Found in late but not early-stage embryos.
	-S	Short	Not found	Not found	Expressed throughout development, not regulated by Elav.
CCND1	A	Short	Nucleus/Cytoplasm in S phase		Can accelerate cell cycle and promote cell proliferation, more stable than CCND1b (conflicting evidence), increased expression
	B	Long	Nucleus	Not found	Can accelerate cell cycle and promote cell proliferation, knockdown promotes apoptosis and suppressed cancer-cell stemness and epithelial mesenchymal transition in human bladder cancer cells.
NET1	Long	Long	Nucleus((more stringently)/Cytoplasm	Not found	Stimulate RhoA activation and actin cytoskeletal rearrangement. Stimulated by Rac1. Preferentially stimulated by estrogen.
	Short	Short	Nucleus/Cytoplasm	Not found	Stimulate RhoA activation and actin cytoskeletal rearrangement. Stimulated by Rac1 heavily to plasma membrane. Significantly promotes gastric cell migration and invasion compared to the long isoform, promotes transcriptional

					activity of reporter gene. Required for proper cell spreading.
IMPA1	L	Long	Distal Axons	Not found	Enriched in distal axons, regulated NGF dependent pathways, involved in survival of sympathetic neuron axons. Suggested main role of providing inositol for recycling of membrane phosphoinisitides.
	S	Short	Proximal Axons	Brain	Enriched in proximal axons
	C	Short	Axons	Brain	Located only in axons, L form cleaved to C form in axons by Hud/Ago2, as efficient as L form at promoting axon integrity
BDNF	Long	Long	Dendrites/Somata	Brain	Long UTR sufficient to target to dendrites, translated locally. Cis-acting translation suppressor at rest. Governs normal dendritic spine development and long-term potentiation. Activity dependent rapid translation from a reporter mRNA in cultured hippocampal neurons.
	Short	Short	Somata	Brain	Mediates active translation to maintain basal levels of BDNF protein-translating polyribosomes in the hippocampus
CD47	Long	Long	Cell Surface	Ubiquitous	Long UTR provides many binding points for HuR RNA binding protein, which has been shown to localize CD47 to the plasma membrane through SET and active RAC1. Cells with high surface levels of CD47 are fully protected from macrophages. This isoform does not affect loss of apoptosis phenotype.
	Short	Short	ER/Cell surface	Ubiquitous	Translocated to ER. Increased susceptibility to macrophage-mediated phagocytosis.
NCAM	120	Short	Ganglion	Brain	Lacks intracellular domain and is attached to the cell via a GPI linkage, most adhesive form. Expression appears to repress metastasis. High expression in ganglion, differentiated neuroblastic tumors.
